# Comparative impacts of polyethylene and biodegradable film residues on soil microbial communities and rapeseed performance under field conditions

**DOI:** 10.3389/fmicb.2025.1553807

**Published:** 2025-05-29

**Authors:** MiaoMiao Xie, Maolu Wei, Qian Sun, Ge Wang, Ting Shen, Xinyi He, Dongyan Liu

**Affiliations:** ^1^Key Laboratory of Land Resources Evaluation and Monitoring in Southwest, Ministry of Education, Sichuan Normal University, Chengdu, China; ^2^College of Life Sciences, Sichuan Normal University, Chengdu, China

**Keywords:** rapeseed, conventional polyethylene films, biodegradable films, soil bacterial community, soil health

## Abstract

**Introduction:**

Soil health is critical for sustainable agriculture and food security, however, the accumulation of agricultural mulch film residues in soil raises environmental concerns. The effects of conventional polyethylene (PE) and biodegradable (PBAT and PLA) film residues on soil health, microbial communities, and crop productivity under field conditions have not been adequately investigated.

**Methods:**

This study simulated the accumulation of PE film residues from over 30 years of continuous mulching and evaluated PBAT and PLA film residues under field conditions, examining their effects on soil physicochemical properties, microbial communities and rapeseed performance.

**Results:**

The results revealed that PE residues significantly altered microbial community composition, enhancing the relative abundance of core genera, including *Sphingomonas*, *Acidibacter,* and *Flavisolibacter*, while suppressing other genera, such as *Burkholderia-Caballeronia-Paraburkholderia*. PE residues also inhibited organic matter decomposition and ureolysis, while limiting nitrate availability and soil fertility, although rapeseed yields remained unaffected. In contrast, biodegradable film residues enhanced soil moisture retention and ammonium content, boosted soil functions such as plastic degradation, nutrient cycling, and chitinolysis, and enriched beneficial genera such as *Candidatus Udaeobacter*, *Acidibacter*, and *Flavisolibacter*, although weakened ureolysis activity. However, both residue types reduced the complexity and stability of the bacterial co-occurrence network, suggesting potential risks to the soil microbial habitats.

**Conclusion:**

These findings demonstrate that conventional film residues had no significant effect on rapeseed productivity, whereas biodegradable films exhibited superior performance in maintaining soil fertility and microbial functions under field conditions. Our study emphasizes the need for long-term monitoring to effectively optimize agricultural plastic film applications.

## 1 Introduction

Agricultural films, particularly white plastic mulches, play a crucial role in agricultural ecosystems. Film mulching boosts crop yields by regulating soil temperature, promoting germination and seedling growth, conserving moisture and accelerating organic matter decomposition. Furthermore, these films improve fertilizer use efficiency, enhance drought resistance, promote earlier crop maturation and ultimately lead to increased yields (Maraveas, 2020; Nizzetto et al., 2016). However, the widespread use of these films, predominantly manufactured from PE, has led to increased soil accumulation owing to inefficient recycling practices and their inherent resistance to degradation (Di Mola et al., 2021). Mechanical stress, weathering, and UV exposure fragment these films into microplastics, introducing persistent pollutants into agricultural ecosystems (Hayes et al., 2017; Kumar et al., 2020; Zhang K. et al., 2021; Song et al., 2023). Mounting evidence suggests that these residues might compromise the soil health and stability (Kumar et al., 2020; Koskei et al., 2021; Lozano et al., 2021; Wang et al., 2021; Huang F. et al., 2023; Zhou et al., 2023).

The microplastics released from agricultural film residues predominantly include fragments, films and fiber forms (Yuan et al., 2022). The current methods for managing these plastic residues in agricultural soils are both time-consuming and costly (Marí et al., 2019). The chemical stability and hydrophobicity of agricultural films render them resistant to biological and chemical degradation, resulting in the accumulation of macro- and microplastics in agricultural soils (Angelucci and Tomei, 2020). Substantial variations in plastic film residue concentrations across different agricultural regions of China have been documented. For instance, concentrations range from 4.94 pieces per kilogram in the lower Yangtze River region to a remarkably high 40,800 pieces per kilogram in Yunnan (Zhang Q. Q. et al., 2021). Among these residues, polyethylene and polypropylene are the predominant (Tang, 2023). This accumulation negatively affects crop productivity through alterations in soil structure and microbial community composition (Zhao et al., 2021; Li C. et al., 2022), ultimately compromising plant growth (Koskei et al., 2021). Yield reductions have been observed across various crops, including wheat (Qi et al., 2020), maize, cotton, potatoes (Gao et al., 2019; Zhang et al., 2020) and green beans (Jiang et al., 2019). In response to these challenges, biodegradable agricultural films have emerged as potential alternatives to conventional PE films (Piyathilake et al., 2024). These films typically degrade within one year in open fields and five years under greenhouse conditions (Velandia et al., 2020). The rapid release of additives also highlight the need for further research to ensure their safe and sustainable application in agriculture.

Soil microbiome underpins soil and human health by mediating nutrient cycling, pollutant degradation and enhancing plant immunity, fertility and yield (Chen et al., 2020; Dai et al., 2020; Wei et al., 2020; Chepsergon and Moleleki, 2023; Liu Z. et al., 2024). The functional capacity of ecosystems, particularly in nutrient cycling processes, is positively correlated with microbial community diversity and network complexity (Wagg et al., 2019; Ling et al., 2022). Recent studies have highlighted the significant influence of microplastics on rhizosphere bacterial communities, including their diversity and abundance (Sun et al., 2021). Both conventional and biodegradable microplastics influence soil nitrogen availability, enzyme activities and diazotrophic networks, with effects varying by type and concentration (Wang et al., 2023). Additionally, microplastics can enhance the rhizosphere microbial abundance and nitrogen metabolism (Kim et al., 2023). These findings are further supported that microplastics significantly affect plant oxidative stress, photosynthetic efficiency and soil microbial activity (Wang W. et al., 2024). PBAT microplastics induce dynamic shifts in bacterial community composition, influenced by particle size, concentration and soil residence time (Shang et al., 2024). Similarly, PLA treatment distinctly alters the bacterial diversity and composition in rice soils compared to PVC and PET (Sun X. et al., 2022). In a rice pot experiment, plastic film residues of different sizes significantly altered soil properties and rhizosphere microbiota, reducing soil density, increasing porosity, modifying enzyme activity and raising dissolved nitrogen levels (Fu et al., 2023). However, research on how conventional and biodegradable agricultural film residues affects soil properties, rhizosphere bacterial communities and plant productivity under field conditions remain limited.

Rapeseed is the predominant oilseed crop in China’s upper Yangtze River region and represents a crucial source of edible oil and renewable energy. The southwestern region alone contributes 28.4% of national production (Hu et al., 2017; Wang H. et al., 2024). This study investigated the effects of PE film residues accumulated over 30 years of continuous mulching, along with one-year PBAT and PLA film residues under field conditions. Specifically, we examined their effects on rapeseed performance, soil physicochemical properties, and microbial communities. We aimed to answer the following three questions: (1) How do these two types of film residue affect rapeseed performance? (2) Do they change soil characteristics, microbial community dynamics? (3) What is the relationship between changes in soil characteristics, microbial community structures and rapeseed productivity?

## 2 Materials and methods

### 2.1 Field design and sample collection

The field experiment was carried out at Qiushi Farm, Chengdu, Sichuan (30°34′N, 104°11′E), as described by Sun et al. (2025). The site is characterized by an annual mean temperature of 14–22°C, average yearly rainfall of 771.8 mm, and a cumulative annual temperature of 4,600–5,000°C. The soil is classified as Alfisols, with a pH ranging from 6.5 to 7.0 and 2–4% organic matter.

**Figure 1:**
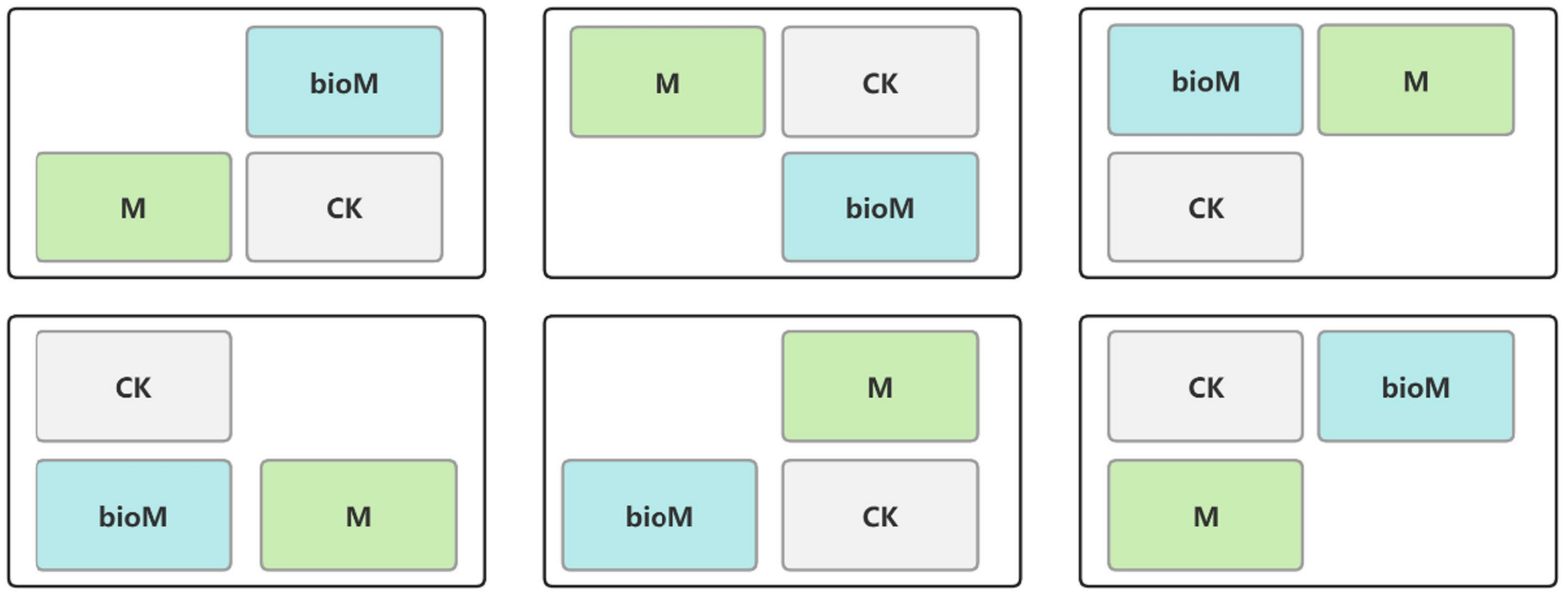
Experimental plot layout diagram.

A 40 × 50 m plot that had functioned as a seedling nursery for the past decade was selected for the experiment. The study used a split-plot design, establishing six main plots (10 × 8 m each), separated by 5 m to reduce edge effects (Figure 1). Each main plot included three treatments: control (CK, no film), PE film addition (M) and PBAT+PLA biodegradable film addition (bioM). The design was based on data regarding residual soil film fragments from 32 years of agricultural film use (Li S. et al., 2022).

Conventional polyethylene (PE) films were purchased from Chengdu Jiuzhou Fengle Agricultural Technology Co., Ltd., while biodegradable PBAT+PLA films were obtained from Jialemi Horticultural Technology Co., Ltd. Both types had a uniform thickness of 0.01 mm. Application rates for film fragments were determined for the 0–30 cm soil layer. For PE films, 0.0915% (w/w) was used for fragments smaller than 4 cm^2^ and 0.0183% (w/w) for fragments between 4–25 cm^2^ (Li S. et al., 2022). For PBAT+PLA films, application rates were based on the actual annual coverage in each plot and prepared fragments accordingly, 83.33 g for pieces smaller than 4 cm^2^ and 41.67 g for those sized 4–25 cm^2^. To simulate natural aging process of film fragments, the experiment began in March 2023 before maize planting. Prior to film fragments were applied, all plots were fertilized with potassium nitrate and urea (N:P:K = 280:112.7:180 g) according to Khan et al. (2017), along with subsequent fertilization as needed (Yousaf et al., 2017). Pre-prepared film fragments were then incorporated into the top 30 cm of soil using a rotary tiller. After the maize harvest in July 2023, soils were tilled and dried to further promote film fragment aging.

In November 2023, rapeseed was sown. Plant and soil samples were collected at three critical stages: 30 days post-transplantation (vegetative stage), 60 days (flowering stage) and 90 days (maturity stage). At each sampling point, both aboveground plant tissues and fine roots were collected for laboratory measurements of physiological traits, including plant height and root diameter, using a steel tape and calipers. Soil samples were taken near plants using a five-point composite sampling method at a depth of 0–20 cm, after removing surface debris. Soil adhering to fine roots was combined to create composite samples, 18 samples per sampling time point, for a total of 54 soil samples over three sampling rounds. In the laboratory, soil samples were passed through a 2 mm sieve and divided: one portion was air-dried for physicochemical analysis, while the other was stored at −80°C for further study. Seed yield was determined by weighing rapeseed after the April 2024 harvest.

### 2.2 Measurement of soil and plant physicochemical parameters

The plant samples were subjected to a two-step drying process: initial blanching at 100°C for 30 min, followed by complete drying at 80°C for moisture content determination. The plant biomass was quantified by direct weighing. For the soil analyses, the moisture content was determined gravimetrically (drying at 105°C), and the pH was measured using a 2.5:1 soil-to-water ratio. The total inorganic nitrogen in the soil was extracted using a 2 M KCl solution. Nitrate nitrogen was quantified using ultraviolet–visible spectrophotometry, whereas ammonium nitrogen was determined using the potassium chloride-indigo blue colorimetric method. Total carbon (TC) was measured using the potassium dichromate method, while total nitrogen (TN) was determined using the Kjeldahl method after digestion with H_2_SO_4_. For soluble organic carbon and nitrogen analysis, samples were extracted with 0.5 M K₂SO₄ (1,5 ratio), followed by quantification procedures similar to those used for total carbon and nitrogen determination.

### 2.3 High-throughput sequencing of bacterial 16S rDNA

Given the consistent physicochemical trends across sampling times, we selected 18 soil samples at the 60-day vigorous growth stage for representative high-throughput sequencing analysis. Genomic DNA was extracted from 0.5–0.7 g soil samples following Liu et al. (2018). DNA quality was verified by 0.7% agarose gel electrophoresis and quantification was performed using a Nanodrop 2000 spectrophotometer. The bacterial 16S rDNA V4 region was amplified using the primers 515F and 806R (Parada et al., 2016). The amplification success was confirmed using 2% agarose gel electrophoresis, followed by sequencing on the Illumina PE250 platform.

### 2.4 Bioinformatics analysis

Raw sequencing data were processed by paired-end merging using the FLASH program, and barcodes were removed during preprocessing. Quality control of the sequences was performed using the QIIME2. Sequences were filtered based on the following criteria: average base quality score <30, length <200 base pairs, or presence of ambiguous bases (N). The Deblur algorithm within QIIME2 was applied for sequence denoising and chimera removal, resulting in Amplicon Sequence Variant (ASV) tables and representative sequences. Taxonomic classification was performed using a Naïve Bayes classifier trained using the SILVA v138 database. Phylogenetic trees were constructed using FastTree plugin QIIME2. To standardize the sampling effort, all samples were rarefied to a minimum sequencing depth of 34,234 reads. All sequence data were deposited in the National Center for Biotechnology Information Sequence Read Archive under accession number PRJNA1197682.

Alpha diversity metrics, including Shannon and Simpson indices, were calculated using the vegan package in R. Comparisons of diversity between treatments were performed using the Wilcoxon rank-sum test for pairwise comparisons and Kruskal-Wallis test for multiple groups, with post-hoc analyses adjusted using the Agricolae package. Beta diversity was assessed using Bray-Curtis distances, which were calculated using the Vegan package in R. Principal coordinate analysis (PCoA) was performed for visualization using the ape and Vegan packages to create ordination plots. Permutational Multivariate Analysis of Variance (PERMANOVA) was performed using the Adonis function in Vegan. To predict potential microbial metabolic and ecological functions, the FAPROTAX tool was used for functional annotation of ASV tables. Random forest analysis was conducted using the RandomForest package in R to identify the key microbial functional groups associated with film residues.

Microbial co-occurrence networks were constructed to evaluate the soil microbial community dynamics. Robust correlations (Spearman correlation coefficients >0.8 or <−0.8 with adjusted *p*-values <0.05) were calculated to define edge connections between nodes (Jiao et al., 2022). Network metrics, including the total number of nodes, links, network diameter, average clustering coefficient, and relative modularity, were computed using the igraph package in R (version 4.3.3) (Yuan et al., 2021). Network visualization was performed using the Gephi software. Positive and negative cohesion values were calculated to assess bacterial community complexity (Herren and McMahon, 2017; She et al., 2021). Network visualizations were generated using the Gephi software. The stability of the soil microbial network was assessed using a network robustness analysis. Simulated random species removal was performed by excluding 50% of the network nodes and evaluating changes in connectivity and network metrics. The ratio of absolute negative to positive cohesion was used to calculate network stability (Wu et al., 2023). Structural equation modeling was conducted to explore causal relationships among soil properties, microbial communities, and rapeseed yield using the plspm package in R (version 4.3.3).

## 3 Results

### 3.1 Fundamental characteristics of plants and soil

**Figure 2:**
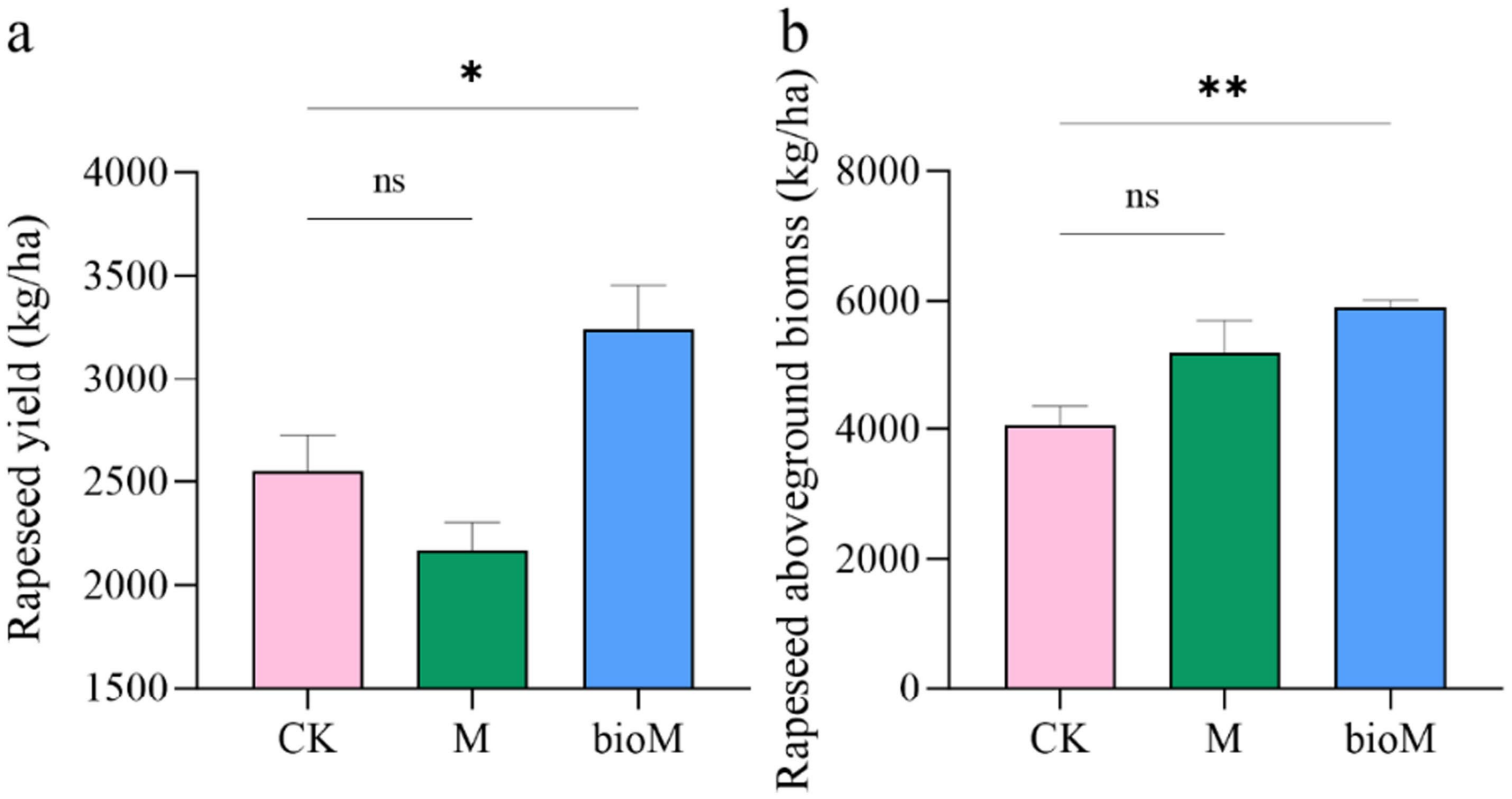
Rapeseed yield **(a)** and aboveground biomass **(b)** in the rhizosphere soil with different treatments. Data are shown as mean ± SE (*n* = 6). An asterisk (*) indicates a significant difference according to the Kruskal-Wallis test with Dunn post-hoc test, with significance levels defined as follows: *p < 0.05, **p < 0.01 and ns indicates no significant difference.

Rapeseed yield and aboveground biomass showed distinct responses to the different treatments (Figures 2a,b). While the M treatment resulted in a slight, non-significant reduction in yield compared to the control (CK), the bioM treatment significantly enhanced both the yield and aboveground biomass (*p* < 0.05). Although the M treatment showed a trend toward increased biomass, this change was not statistically significant. The soil physicochemical properties were substantially altered by both treatments (Table 1). The M treatment significantly increased soil pH and total carbon compared to CK (*p* < 0.05), but decreased inorganic nitrogen (IN) and total nitrogen content (*p* < 0.05). Although not statistically significant, reductions were observed in NH_4_^+^-N, NO_3_^−^-N, dissolved organic carbon (DOC), and dissolved organic nitrogen (DON). In contrast, the bioM treatment significantly enhanced soil moisture content, NH_4_^+^-N, NO_3_^−^-N, IN, DOC and total carbon compared with CK (*p* < 0.05).

**Table 1:** Physiochemical properties in rhizosphere soil with different treatments.

Parameter	CK	M	bioM
pH	6.51 ± 0.05b	6.82 ± 0.04a	6.57 ± 0.09b
SMC(%)	12.06 ± 0.25c	13.27 ± 0.35b	14.00 ± 0.43a
NH_4_^+^-N(mg/kg)	3.09 ± 0.10b	2.94 ± 0.02b	3.64 ± 0.12a
NO_3_^−^-N(mg/kg)	12.96 ± 0.85b	11.67 ± 0.78b	18.30 ± 1.46a
IN(mg/kg)	16.05 ± 0.85b	14.61 ± 0.80c	21.94 ± 1.42a
DOC(g/kg)	16.46 ± 0.99b	15.70 ± 1.15b	19.72 ± 1.97a
DON(g/kg)	0.25 ± 0.01a	0.24 ± 0.00a	0.43 ± 0.00a
TC(g/kg)	41.42 ± 0.48c	43.67 ± 0.47b	45.53 ± 0.81a
TN(g/kg)	2.77 ± 0.16a	2.42 ± 0.02b	2.84 ± 0.03a

Data are shown as mean ± SE (*n* = 6). Different letters indicate significant difference (*p* < 0.05) according to the Kruskal-Wallis test with Dunn post-hoc test among the treatments. SMC, soil water content; NH_4_^+^-N, soil ammonium; NO_3_^−^-N, soil nitrate; IN, inorganic nitrogen; DOC, dissolved organic carbon; DON, dissolved organic nitrogen; TC, total carbon; TN, total nitrogen.

### 3.2 Diversity and structure of soil bacterial communities

**Figure 3:**
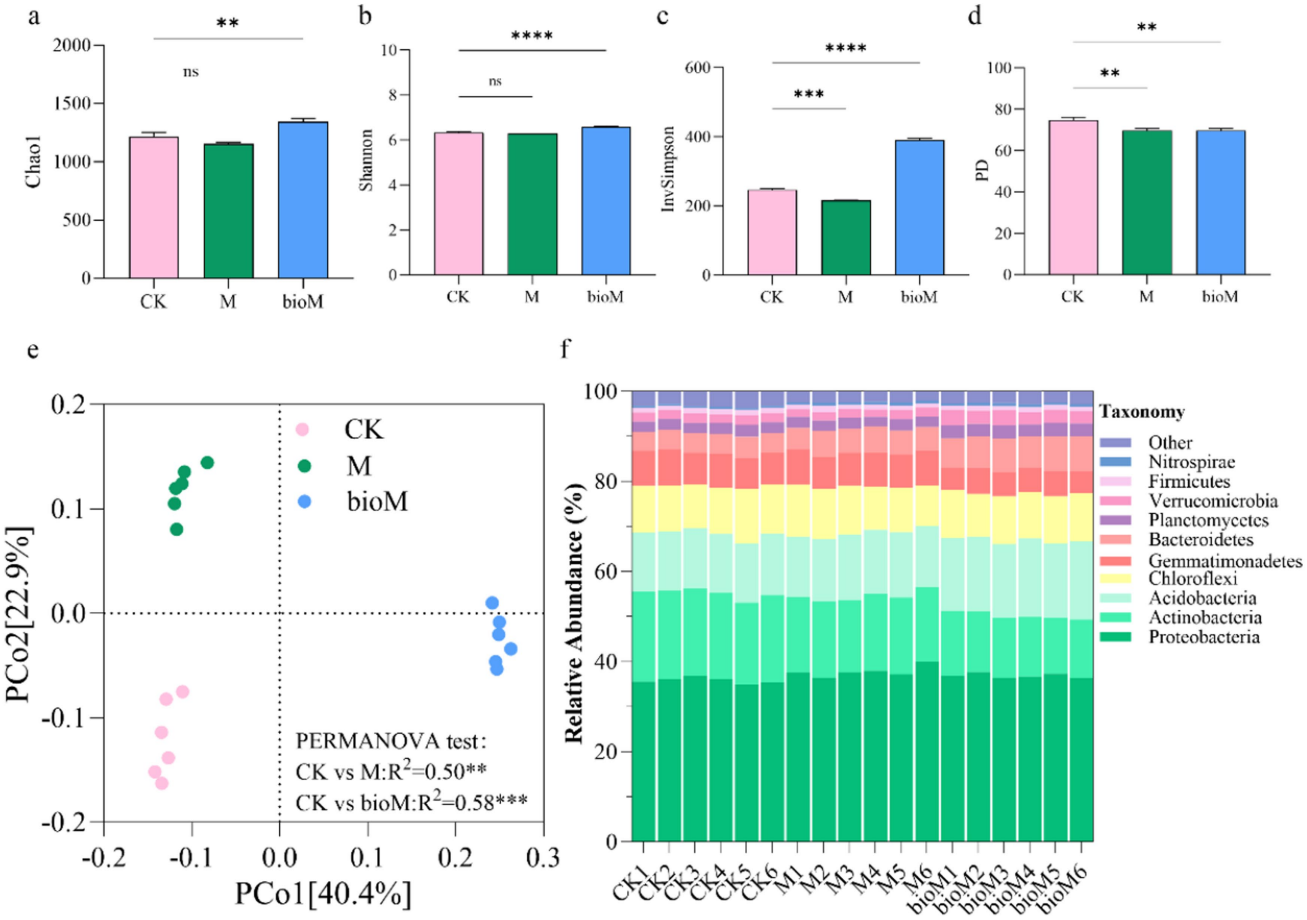
Alpha diversity indices of Chao1 **(a)**, Shannon **(b)**, Simpson **(c)**, PD **(d)**, PCoA analysis based on Bray-Curtis distance **(e)** and relative abundance of top 10 phyla **(f)** of bacterial community in rhizosphere soil with different treatments. Data are shown as mean ± SE (*n* = 6). An asterisk (*) indicates a significant difference according to the Kruskal-Wallis test with Dunn post-hoc test, with significance levels defined as follows: **p < 0.01, ***p < 0.001, ****p < 0.0001 and ns indicates no significant difference.

High-throughput sequencing at 100% similarity identified 6,268 bacterial Amplicon Sequence Variants (ASVs), with 1,467, 1,212, and 1729 unique ASVs in CK, M, and bioM treatments, respectively. Among these, 1,038 ASVs were shared across all the treatments (Supplementary Figure S1). Diversity indices (Chao1, Shannon and InvSimpson) consistently showed that the bioM treatment significantly enhanced bacterial diversity compared to CK, while the M treatment reduced diversity (Figures 3a–c). However, both M and bioM treatments significantly reduced Phylogenetic Diversity compared to CK (Figure 3d). Principal coordinate analysis (PCoA) revealed distinct bacterial community compositions among the treatments (Figure 3e). The first two PCoA axes explained 63.3% of the total variance (PC1, 40.4%; PC2, 22.9%). PERMANOVA confirmed significant differences among all treatments: CK vs. M (R^2^ = 0.50, *p* = 0.003), CK vs. bioM (R^2^ = 0.58, *p* = 0.001) and M vs. bioM (R^2^ = 0.59, *p* = 0.002).

The top 10 bacterial phyla maintained similar compositions across treatments, although their relative abundances varied considerably (Figure 3f, Supplementary Figure S2). Proteobacteria was dominant across all plots, followed by Actinobacteria, Acidobacteria, Chloroflexi, Gemmatimonadetes, and Bacteroidetes. The M treatment showed increased relative abundances of Proteobacteria, Acidobacteria, Bacteroidetes, and Nitrospirae, but decreased Actinobacteria compared to CK. BioM treatment enhanced the relative abundance of Acidobacteria, Bacteroidetes, Planctomycetes, Verrucomicrobia, and Nitrospirae while reducing the abundance of Actinobacteria and Gemmatimonadetes.

**Figure 4:**
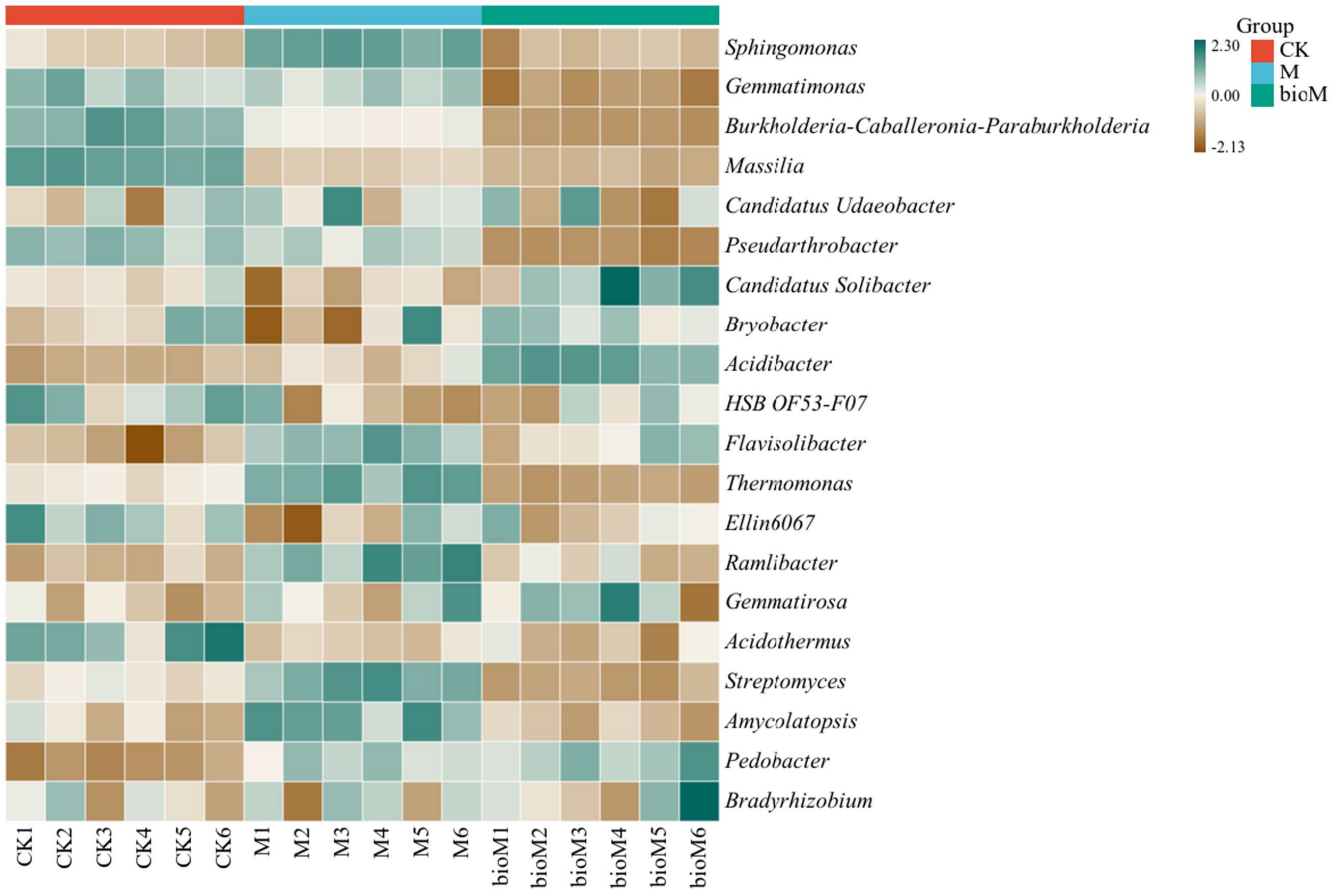
Heatmap of top 30 genera of the bacterial community in rhizosphere soils with different treatments.

At the genus level, both treatments induced distinct community shifts (Figure 4, Supplementary Figure S3). M significantly increased the relative abundances of *Sphingomonas*, *Acidibacter*, *Flavisolibacter*, *Thermomonas*, *Ramlibacter*, *Streptomyces*, *Amycolatopsis* and *Pedobacter*, while reducing *Burkholderia*-*Caballeronia*-*Paraburkholderia*, *Massilia*, *Pseudarthrobacter*, HSB OF53-F07, Ellin6067 and *Acidothermus*. BioM treatment enhanced *Candidatus Udaeobacter*, *Acidibacter*, *Flavisolibacter* and *Pedobacter*, while decreasing *Gemmatimonas*, *Burkholderia*-*Caballeronia*-*Paraburkholderia*, *Massilia*, *Pseudarthrobacter*, *Thermomonas*, *Acidothermus* and *Streptomyces* compared to CK.

### 3.3 Prediction of soil bacterial community functions

**Figure 5:**
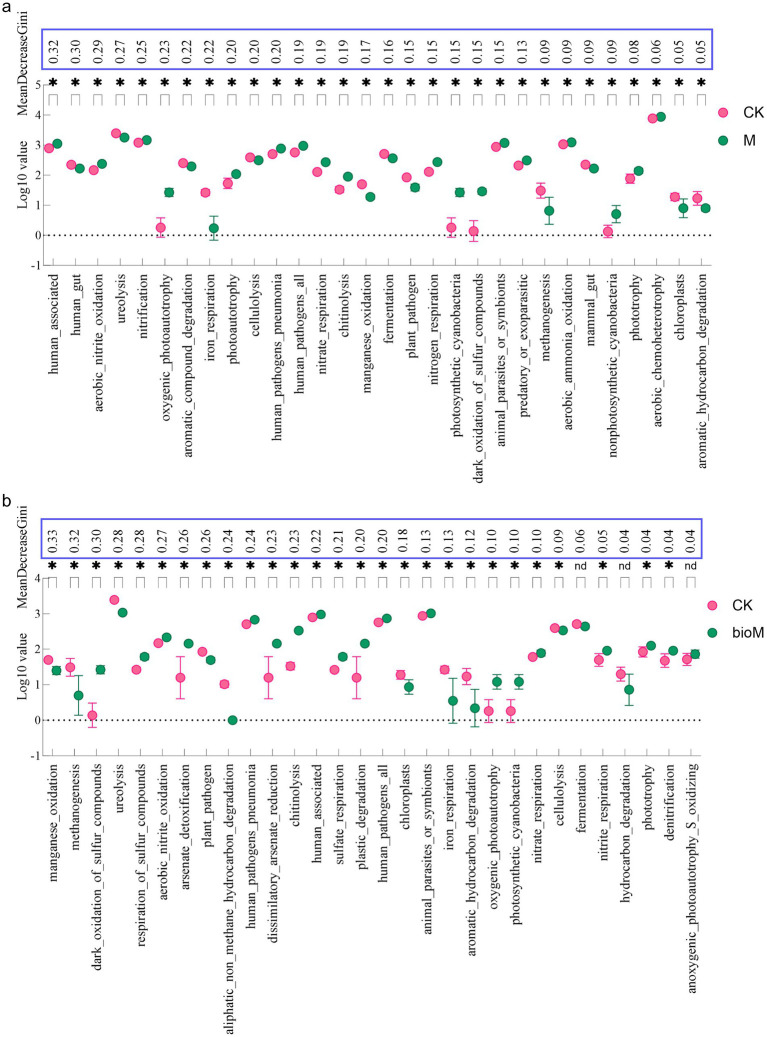
Random Forest analysis of top 30 bacterial functional groups based on FAPROTAX analysis between CK vs. M **(a)** and CK vs. bioM **(b)** treatments.

FAPROTAX analysis of 6,268 bacterial ASV sequences identified 92 functional groups, encompassing metabolic processes related to nutrient cycling (nitrogen, carbon, and sulfur), metal transformations (As and Fe), and ecological interactions. Random forest analysis of the 30 key functional groups revealed treatment-specific variations in carbon and nitrogen cycling (Figures 5a,b). In the M treatment, carbon-related functions including oxygenic photoautotrophy, phototrophy, and aerobic chemoheterotrophy showed significant increases compared with CK. However, functions related to aromatic compound degradation, cellulolysis, fermentation, methanogenesis, and chloroplast processes were reduced. Nitrogen-related functions, including aerobic nitrite oxidation, nitrification, nitrate respiration, and chitinolysis, were significantly enhanced, while ureolysis decreased (Figure 5a). The bioM treatment significantly enriched carbon functional groups associated with chitinolysis, plastic degradation, and photosynthetic processes (oxygenic photoautotrophy, phototrophy, and photoautotrophy) compared to CK, while reducing methanogenesis, hydrocarbon degradation, cellulolysis, and aromatic compound degradation. Similarly, nitrogen cycling functions such as aerobic nitrite oxidation, nitrate respiration, and nitrite denitrification were enhanced, although ureolysis decreased (Figure 5b).

### 3.4 Co-occurrence network of soil bacterial communities

**Figure 6:**
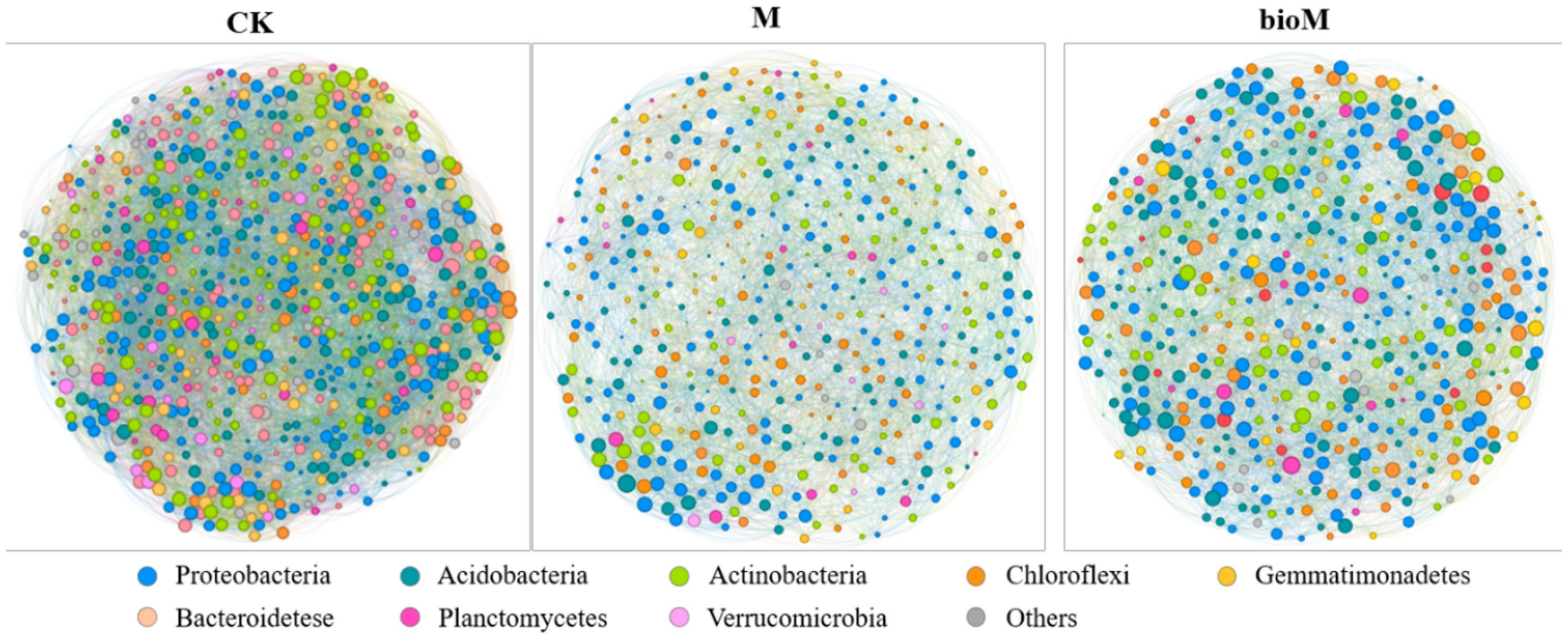
The co-occurrence networks of soil bacterial communities at the ASV level in rhizosphere soil with different treatments.

**Figure 7:**
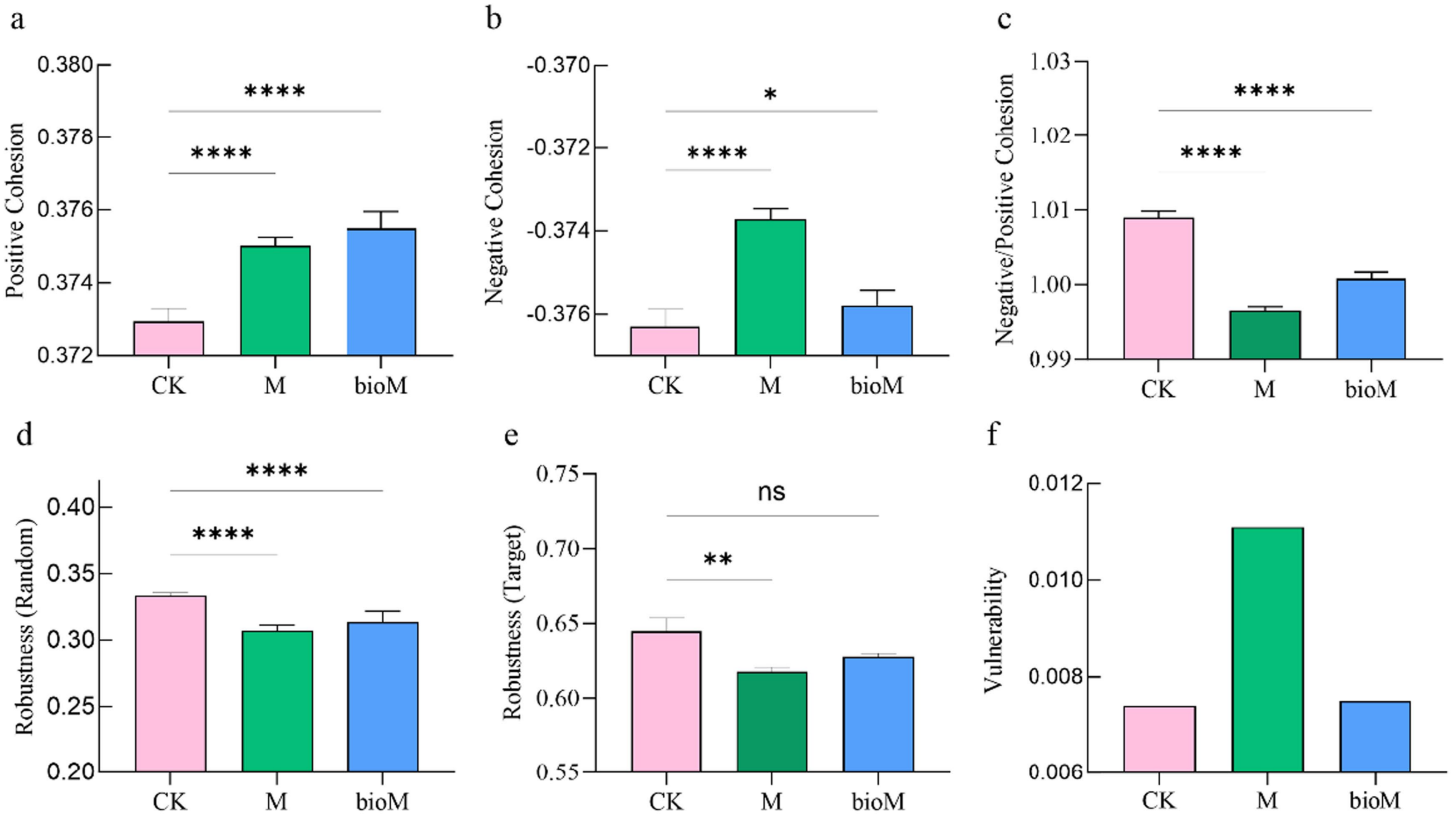
Microbial network stability indices, including positive cohesion **(a)**, negative cohesion **(b)**, network complexity **(c)** robustness after random removal of samples **(d)**, robustness after targeted removal of specific samples **(e)** and vulnerability **(f)** in the rhizosphere soil with different treatments. Data are shown as mean ± SE (*n* = 6). An asterisk (*) indicates a significant difference according to the Kruskal-Wallis test with Dunn post-hoc test, with significance levels defined as follows: *p < 0.05, **p < 0.01, ****p < 0.0001 and ns indicates no significant difference.

Network analysis revealed distinct structural changes across the treatments (Figure 6; Table 2). Both M and bioM treatments showed reduced network complexity compared with CK, with lower modularity (M: 0.445, bioM: 0.459, CK: 0.497), average degree (M: 27.28, bioM: 29.41, CK: 43.03)and clustering coefficients (M: 0.417, bioM: 0.409, CK: 0.428). Conversely, the average path lengths (M: 0.019, bioM: 0.020, CK: 0.011) and network diameters (M = 0.041, bioM = 0.038, CK = 0.025) increased significantly. While positive and negative cohesive forces were strengthened in both M and bioM treatments compared to CK (Figures 7a,b), network complexity decreased (Figure 7c). Random node removal analysis demonstrated reduced network robustness for both treatments (Figures 7d,e). The M treatment showed the highest bacterial network vulnerability, while bioM and CK maintained similar vulnerability indices (Figure 7f).

**Table 2:** Topological properties of network in rhizosphere soil with different treatments.

Parameter	CK	M	bioM
Nodes	731	458	501
Edges	15,726	6,247	7,367
Positive correlation	7,855	3,106	3,686
Negative correlation	7,871	3,141	3,681
Average degree	43.03	27.28	29.41
Network density	0.059	0.060	0.059
Clustering coefficient	0.43	0.42	0.41
Average path length	0.011	0.019	0.020
Network diameter	0.025	0.041	0.038
Modularity	0.50	0.46	0.46

### 3.5 PLS-PM model of bacterial communities

**Figure 8:**
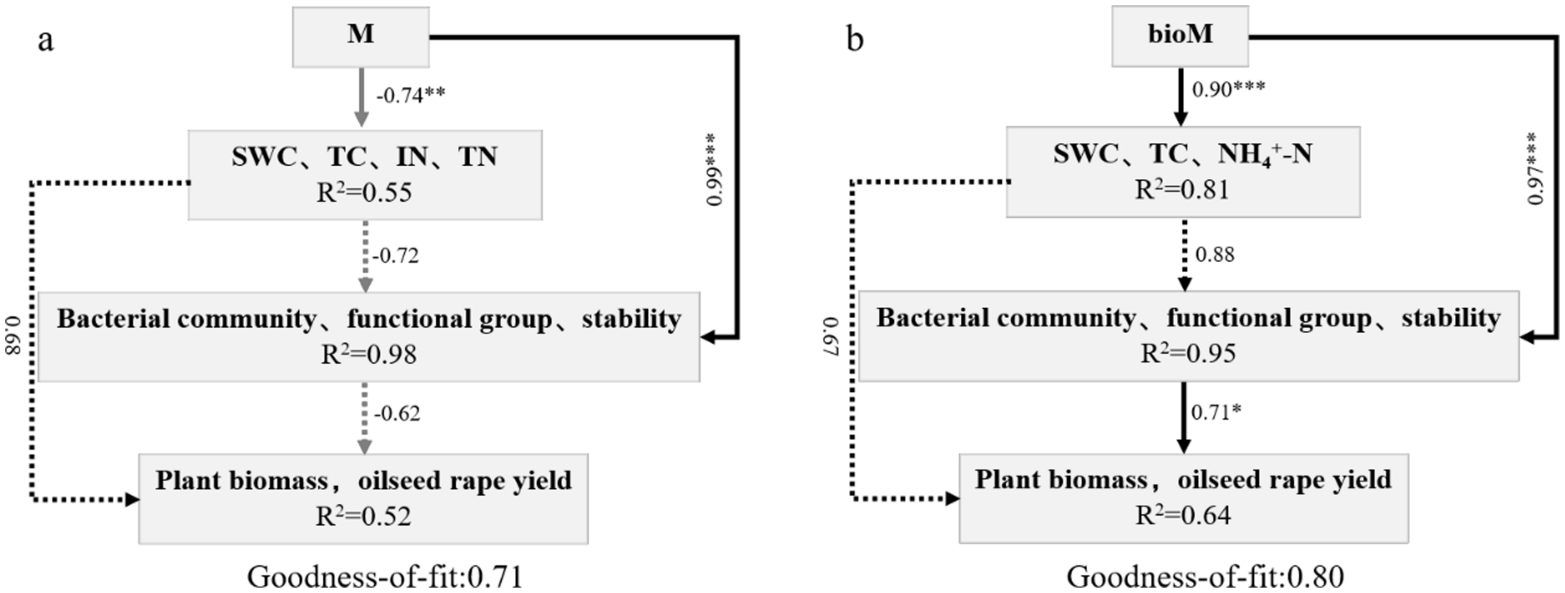
PLS-PM analysis the effects of soil abiotic factors, soil bacterial community, functional group and network stability on rapeseed growth in M **(a)** and bioM **(b)** treatments. The path coefficients are indicated by the numbers adjacent to the arrows. Solid arrows represent significant path coefficients (p < 0.05), while dashed arrows indicate non-significant coefficients (*p* > 0.05). Positive and negative influences are represented in black and gray, respectively.

The partial least squares path model (PLS-PM) revealed distinct treatment effects on soil–plant-microbe interactions (Figure 8). The M treatment model (goodness of fit: 0.71) showed strong positive effects on bacterial communities, functions, and network stability (*r* = 0.99), but negatively affected soil properties, particularly nitrogen content (*r* = −0.74), with no direct effect on rapeseed biomass or yield (Figure 8a). The bioM treatment model (goodness of fit: 0.80) demonstrated more comprehensive positive effects, enhancing bacterial communities, functions, and network stability (*r* = 0.97), which directly improved the rapeseed biomass and yield (*r* = 0.91). Additionally, bioM treatment positively influenced soil properties (*r* = 0.90), creating an indirect positive feedback loop through bacterial communities (*r* = 0.88), which further enhanced plant performance (*r* = 0.71) (Figure 8b).

## 4 Discussion

In this study, the effects of conventional PE and biodegradable PBAT-PLA film residues on bacterial communities, soil properties and rapeseed performance were comprehensively analyzed. All experimental questions were confirmed. Conventional PE residues had no significant impact on rapeseed biomass or yield but decreased soil inorganic nitrogen, bacterial *α*-diversity, and organic matter degradation functions, significantly weakening microbial network stability. In contrast, biodegradable residues enhanced rapeseed productivity by improving soil fertility and microbial diversity and enriching functional groups involved in carbon and nitrogen cycling, although they also moderately reduced microbial network stability.

### 4.1 Impact of agricultural film residues on plant performance and soil physicochemical properties

Our study compared the ecological impacts of conventional polyethylene (PE) and biodegradable PBAT-PLA mulch films, revealing distinct effects on plant growth and soil properties (Figure 2; Table 1). Recent studies have highlighted the significant effects of agricultural film residues on plant growth and soil microbial communities (Qi et al., 2022; Zhou et al., 2023; Shirin et al., 2024; Yang et al., 2024). While previous research has documented the negative effects of film residues on various crops including wheat, maize, potato and cotton (Gao et al., 2019; Qi et al., 2020; Zhang et al., 2020), our findings showed treatment-specific responses.

PE film residues had minimal impact on rapeseed biomass and yield, consistent with observations that PE microplastics negligibly affected maize growth (Ding et al., 2023). In contrast, PBAT-PLA residues significantly enhanced both parameters, aligning with reports of increased peanut yields under biodegradable mulch films (Zhao et al., 2023). One key reason for the difference between our results and those of previous studies is the concentration of film residues applied (Qi et al., 2018). Our experiment applied much lower residue levels, about 0.1% w/w for PE and similarly low rates for PBAT-PLA. In contrast, Qi et al. (2018) used a much higher concentration of 1%. Their study found negative effects at this level, no matter the fragment size. This highlights a strong dose-dependent effect. For example, Zhang et al. (2020) reported that with every 100 kg ha^−1^ increase in residual mulch film, maize plant height decreased by 2.5%. Therefore, the lower and more realistic concentrations used in our study may explain why we did not observe the same adverse effects as those reported with higher doses.

The observed changes in plant performance were correlated with alterations in soil physicochemical properties, which are crucial determinants of bacterial function (Wu et al., 2022). Both film types significantly increased the total soil carbon content, consistent with previous studies showing enhanced soil organic matter and carbon content following microplastic addition (Kim et al., 2021). For instance, high concentrations (28%) of polypropylene microplastics have been shown to increase soil soluble organic carbon by over 35% (Liu et al., 2017). This suggests that plastic mulch residues contribute to soil carbon content independently of photosynthetic processes and net primary production (Rillig and Lehmann, 2020), although distinguishing between soil and plastic-derived carbon remains technically challenging.

The treatments had contrasting effects on soil nitrogen dynamics. PBAT-PLA films significantly increased inorganic nitrogen (ammonium and nitrate) and soluble organic nitrogen concentrations, while PE films reduced inorganic nitrogen levels. These findings align with previous research showing that non-degradable plastics (0.3–1%) reduce nitrate and ammonium nitrogen in rice paddy soils (Sun X. et al., 2022). Such changes in nitrogen availability directly influence crop performance, as demonstrated by studies showing reduced maize growth and nitrogen uptake in soils containing 0.5% PET microplastics (Ingraffia et al., 2022; Hu et al., 2020). The differential effects of plastic type on soil nitrogen cycling processes are well documented (Seeley et al., 2020), which enhances nitrification and denitrification, while polyvinyl chloride inhibits these processes. Our results support these findings, demonstrating that biodegradable films improve soil nutrient status and consequently enhance rapeseed yield.

### 4.2 Influence of agricultural film residues on soil bacterial community diversity and composition

Agricultural film residues significantly influenced soil bacterial *α*-diversity, depending on the treatment (Figure 3). BioM treatment enhanced bacterial α-diversity compared to the controls, which is consistent with previous observations of increased microbial diversity under biodegradable film treatments (Lian et al., 2022). Conversely, conventional PE films (M treatment) reduced bacterial α-diversity, aligning with studies showing decreased bacterial abundance and diversity following the addition of 1% LDPE addition (Fei et al., 2020). This divergent effect on bacterial diversity likely stems from differences in carbon bioavailability between film types. Biodegradable plastics hydrolyze into water-soluble, low-molecular-weight oligomers that serve as readily available carbon sources for microorganisms (Sun Y. et al., 2022). The increased soil organic nitrogen and carbon levels observed in bioM treated soils were positively correlated with enhanced microbial diversity (Li C. et al., 2022), suggesting that improved nutrient availability supports more diverse bacterial communities.

Both treatments significantly altered the composition of the dominant bacterial taxa, reflecting the strong relationship between soil nutrient status and bacterial diversity (Wagg et al., 2021). The predominant phyla identified were Proteobacteria, Actinobacteria, Acidobacteria, Chloroflexi, Gemmatimonadetes, and Bacteroidetes, which is consistent with communities found in organically enriched and microplastic-contaminated soils (Li C. et al., 2022). The M treatment enhanced the abundance of Proteobacteria, Acidobacteria, Bacteroidetes, and Nitrospirae abundance while reducing Actinobacteria. The bioM treatment increased Acidobacteria, Bacteroidetes, Planctomycetes, Verrucomicrobia and Nitrospirae, but decreased Actinobacteria and Gemmatimonadetes (Figure 3, Supplementary Figure S2). These shifts reflect the functional roles of specific taxa. Proteobacteria, among the most metabolically versatile phyla, contribute to various physiological processes and nutrient cycling, including the degradation of biodegradable PBAT and PLA films (Han et al., 2021). Acidobacteria serves as an indicator of soil nutrient status (Acuña et al., 2020), while Bacteroidetes thrives in nutrient-rich environments, facilitating nutrient cycling (Griffith et al., 2017). Actinobacteria, which are crucial for rhizosphere processes, support nutrient cycling and promote plant growth (Merzaeva and Shirokikh, 2006).

At the genus level (Figure 4, Supplementary Figure S3), conventional film residues enhanced populations of hydrocarbon-degrading bacteria such as *Sphingomonas*, Streptomyces, and *Amycolatopsis*, which promote soil health through nutrient cycling and organic matter decomposition (Chigwada and Tekere, 2023; Shen et al., 2023; Zhou et al., 2024). *Sphingomonas* specializes in complex organic compound metabolism and pollutant degradation (Aulestia et al., 2021). The observed decrease in *Burkholderia*-*Caballeronia*-*Paraburkholderia*, *Massilia* and *Acidothermus* likely reflects habitat disruption by film residues, potentially affecting their roles in nitrogen assimilation and plant-microbe interactions (Liu R. et al., 2024; Zhang et al., 2024). In contrast, biodegradable film residues enriched *Candidatus Udaeobacter*, *Acidibacter*, *Flavisolibacter* and *Pedobacter* genera, suggesting enhanced organic carbon and nutrient availability. These genera play crucial roles in organic matter degradation and nutrient mobilization and support plant growth and soil fertility (Brabcova et al., 2016; Li C. et al., 2022).

### 4.3 Impact of agricultural film residues on predicted functional profiles of soil bacterial communities

FAPROTAX analysis revealed treatment-specific effects on bacterial functional profiles (Figures 5a,b), with primary functions centered on nitrogen and carbon cycling, consistent with recent studies (Liu et al., 2023). Soil microbial communities are crucial mediators of nutrient cycling, particularly for nitrogen and carbon transformations (Liang et al., 2020). In the M treatment, carbon-related functional genes showed divergent responses. While genes associated with oxygenic photosynthesis and phototrophic processes increased significantly, those involved in aromatic hydrocarbon and cellulose degradation decreased compared to controls. This suggests that conventional PE film residues may impede organic carbon transformation and energy release from organic matter, potentially compromising soil ecological function and agricultural productivity (Qian et al., 2018). The inert nature and slow turnover of conventional film residues likely restrict organic carbon availability for microbial utilization. The nitrogen cycling genes in the M treatment exhibited complex dynamics. Despite enrichment in groups related to aerobic nitrous oxide oxidation, nitrification and nitrogen respiration, urease-related functions decreased significantly. This imbalance may impair nitrogen mineralization and plant nitrogen uptake, which is consistent with previous observations that microplastic addition (1% LDPE, 30-day exposure) reduces nitrate reduction functionality (Chen et al., 2022).

The bioM treatment demonstrated distinct functional patterns, with significant enrichment of groups related to chitin degradation, plastic degradation and photoautotrophy, while showing reduced abundance of non-methane aliphatic and aromatic hydrocarbon degradation groups. These changes suggest optimized organic carbon transformation and resource utilization, potentially contributing to improved agricultural yields (Huang J. et al., 2023). Nitrogen cycling groups in the bioM treatment showed a broad enhancement, despite the reduced urease-related group abundance. This pattern indicates that biodegradable film residues may provide nutrient-rich substrates that support increased microbial biomass and enhanced nitrogen cycling capacity (Zhang et al., 2024). Such improvements in microbial performance can enhance nitrogen fixation and plant nutrient uptake, ultimately promoting plant growth and crop yields (Tang et al., 2020).

The influence of agricultural film residues, particularly biodegradable variants, extends beyond the immediate nutrient cycling. These materials can reshape microbial community structure and function by providing energy and nutrients that support microbial proliferation and enzyme activity (Sun Y. et al., 2022). However, the observed enrichment of human pathogens and animal parasites in both treatments warrant careful consideration. Although FAPROTAX provides valuable insights, further research is needed to validate these functional predictions and their implications for agricultural systems.

### 4.4 Impact of agricultural film residues on co-occurrence network stability of soil bacterial communities

Both conventional and biodegradable film residues significantly altered soil bacterial co-occurrence networks, reducing their complexity and stability (Figures 6, 7). The observed decrease in network nodes and connections indicates diminished interconnectedness within the microbial communities. Cohesion parameter analysis, which characterizes the strength of positive and negative bacterial relationships and overall community stability (Yuan et al., 2021), revealed that both film types, particularly conventional films, reduce bacterial community complexity and potential species interactions. Network modularity, an indicator of distinct functional habitats within microbial communities (Hernandez et al., 2021), decreased by 10 and 8% in the conventional and biodegradable film treatments, respectively. This reduction suggests the loss of functional niches in response to agricultural film residues (Yuan et al., 2021; Shi et al., 2022), potentially compromising ecosystem functionality. The observed decrease in network stability indicates reduced resistance to external perturbations, which typically correlates positively with the network complexity (Cornell et al., 2023; Artime et al., 2024). This diminished modularity further suggests limited ecosystem resilience, consistent with previous studies showing that residual agricultural films disrupt co-occurrence networks and alter microbial diversity (Wu et al., 2022). The decreased network robustness, particularly pronounced in conventional film treatments, indicates increased vulnerability of microbial communities to environmental stressors.

While both film types affected network stability, biodegradable films appeared to have less severe effects. Their potential to serve as labile carbon sources may promote microbial metabolic activity and enhance interactions related to resource utilization and niche differentiation compared with conventional films (Huang F. et al., 2023). However, the long-term implications of biodegradable film residues, particularly their accumulation and impact on soil ecosystem stability, require further investigation (Zhu et al., 2022; Rillig et al., 2024).

## 5 Conclusion

This study compared the effects of conventional PE film residues and biodegradable PBAT-PLA residues on plant performance, soil properties and microbial communities under field conditions. Conventional PE film residues had minimal impact on rapeseed biomass and yield, but significantly reduced inorganic and total nitrogen contents, thereby impairing soil fertility. They also decreased the abundance of Actinobacteria, potentially hindering organic matter degradation by suppressing key functions such as aromatic hydrocarbon and cellulose decomposition. Furthermore, PE residues substantially weaken microbial network stability and increase the ecosystem vulnerability to external stressors. In addition, biodegradable PBAT-PLA film residues significantly enhanced rapeseed biomass and yield by improving soil fertility and microbial diversity. These residues enriched the functional groups associated with carbon and nitrogen cycling, such as those involved in chitin and plastic degradation, nitrogen respiration, optimizing nutrient cycling and resource utilization. While biodegradable residues also reduced microbial network stability, their effects were less disruptive than those of PE residues. In summary, although biodegradable films offer short-term benefits for crop productivity and soil fertility, both conventional and biodegradable residues negatively impact soil microbial community structure and stability. These findings highlight the need to consider the long-term ecological effects of all types of film residues when promoting agricultural plastic alternatives.

## Data Availability

The datasets presented in this study can be found in online repositories. The names of the repository/repositories and accession number(s) can be found in the article/Supplementary material.

## Supplementary material

The Supplementary material for this article can befound online at: https://www.frontiersin.org/articles/10.3389/fmicb.2025.1553807/full#supplementary-material
