# Supplementary material for: Comparative impacts of polyethylene and biodegradable film residues on soil microbial communities and rapeseed performance under field conditions
Source: Front Microbiol. 2025 May 29;16:1553807. doi: 10.3389/fmicb.2025.1553807 (PMC12159761; doi:10.3389/fmicb.2025.1553807)
Supplement: Supplementary file 1 [file Supplementary_file_1.docx]

Comparative impacts of polyethylene and biodegradable film residues on soil microbial communities and rapeseed performance under field conditions

MiaoMiao Xie^1,2^, Maolu Wei^1,2^，Qian Sun^1,2^, Ge Wang^1,2^，Ting Shen^1,2^，Xinyi He^1,2^, Dongyan Liu^1,2,*^

^1^ Key Laboratory of Land Resources Evaluation and Monitoring in Southwest (Sichuan Normal University), Ministry of Education, Chengdu 610101, China

^2^ College of Life Sciences, Sichuan Normal University, Chengdu, 610041, China;

* Correspondence: [liudy@sicnu.edu.cn (DY.Liu)](mailto:liudy@sicnu.edu.cn%20(DY.Liu))

Fig S1. Venn diagram of CK, M, and Bio-M agricultural film residue treatments in rhizosphere soil.


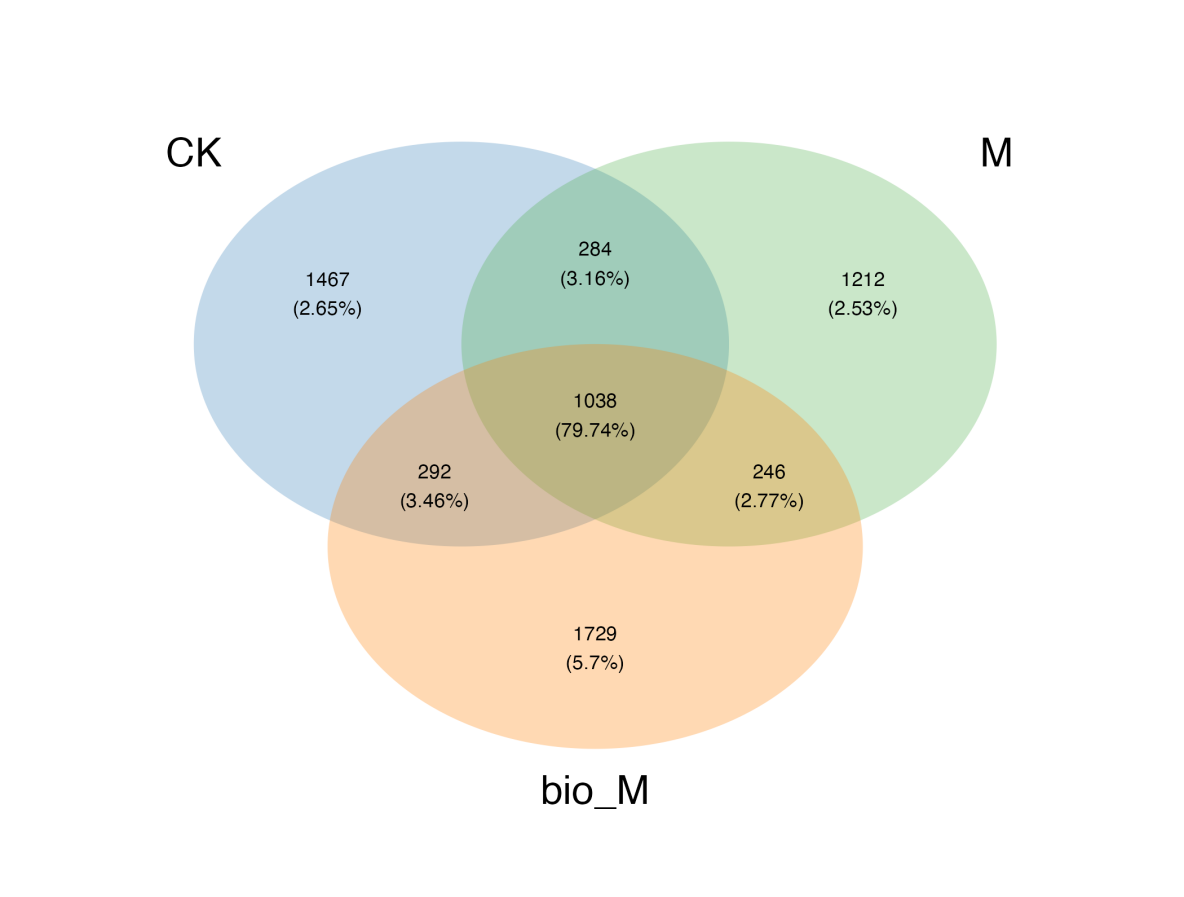

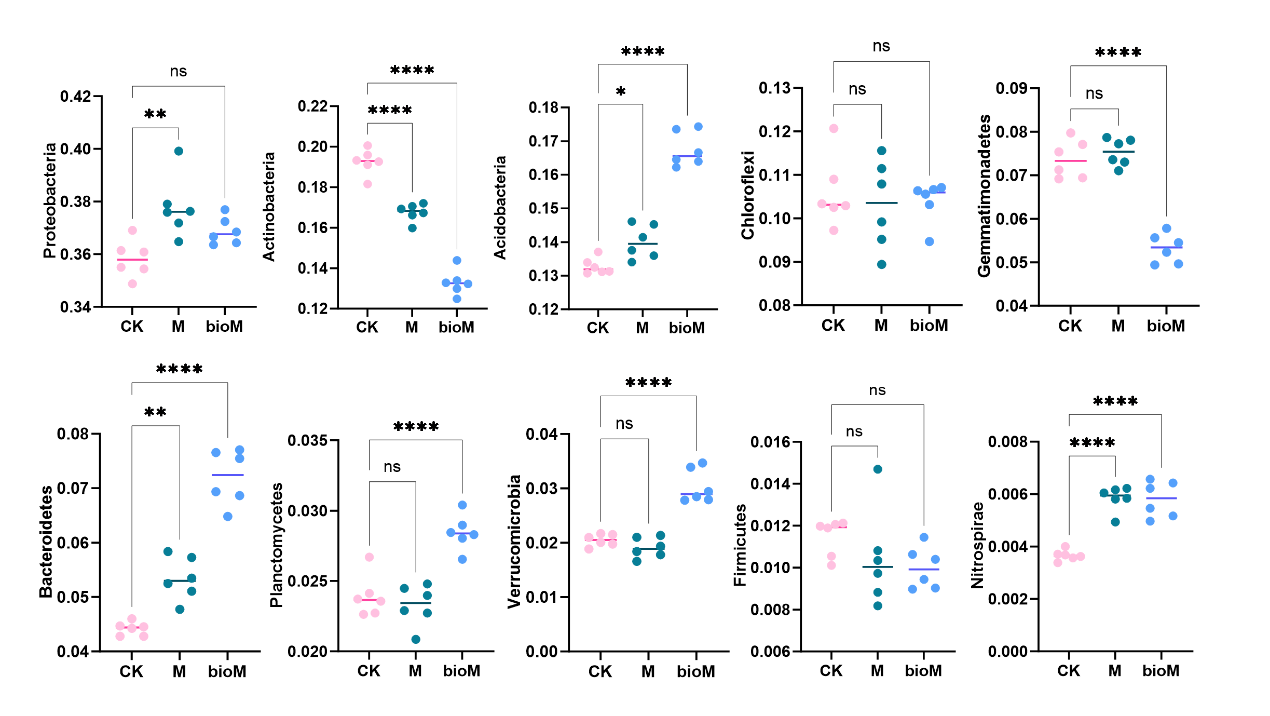


Fig S2. Comparison of the relative abundance of various bacterial phyla under different treatment groups (CK, M, and Bio-M) using the Kruskal-Wallis test. The figure displays the log-transformed values plus one (Log10-transformed value) for each phylum. The left side shows the abundance data for each phylum, with boxes representing data points for the different treatment groups and error bars indicating standard error. The right column presents the adjusted p-values from the Dunn test, indicating the significance of differences between treatment groups. The blue, red, and green boxes represent the CK, M, and Bio-M treatment groups, respectively; different p-values denote the significance of differences between treatment groups, with p < 0.05 considered significant.


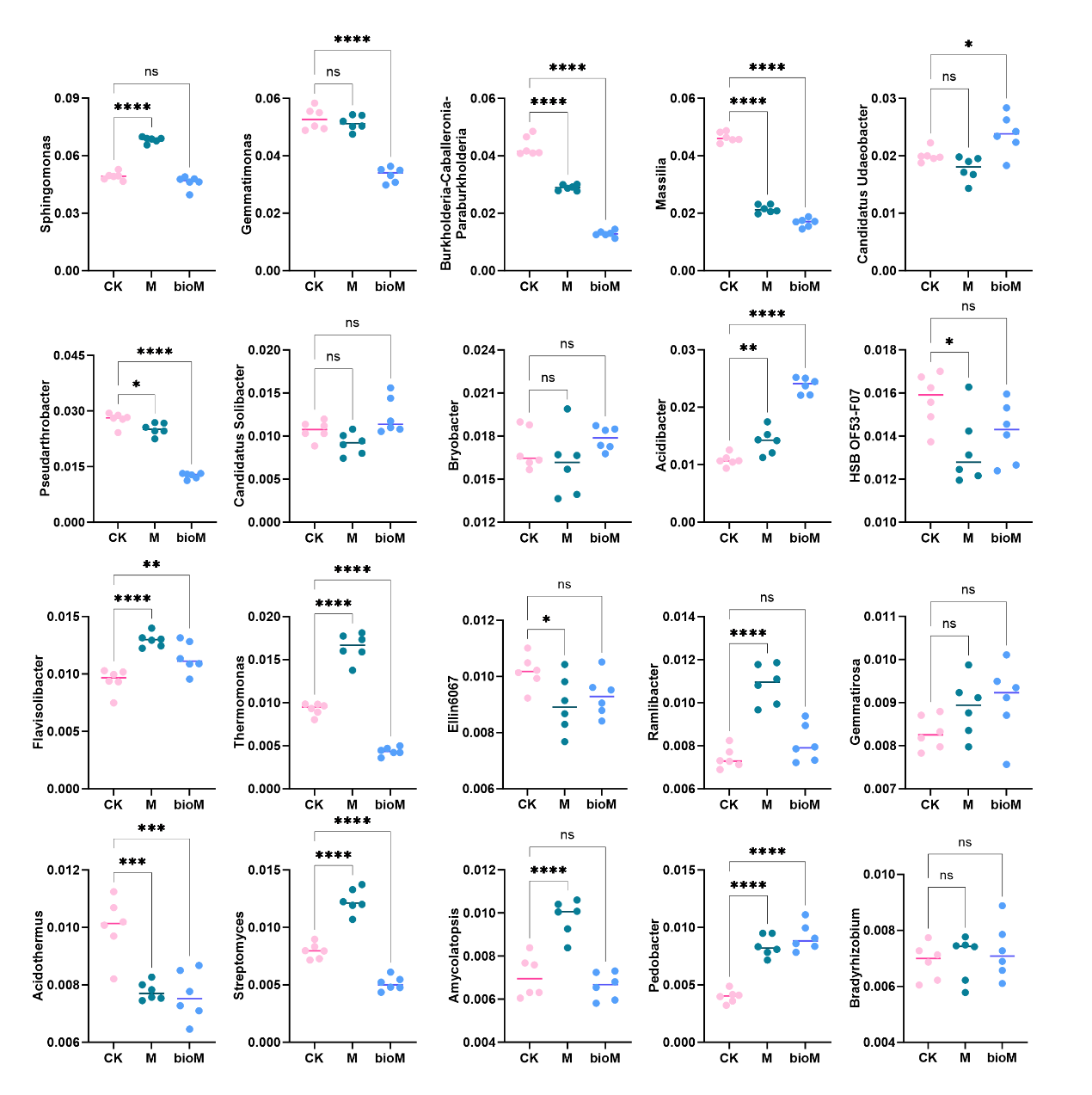


Fig S3. Comparison of the relative abundance of various bacterial genera under different treatment groups (CK, M, and Bio-M) using the Kruskal-Wallis test and Dunn test. The figure displays the log-transformed values plus one (Log10-transformed value) for each genus. The left side shows the abundance data for each genus, with boxes representing data points for different treatment groups and error bars indicating standard error. The right column presents the adjusted p-values from the Dunn test, indicating the significance of differences between treatment groups. The blue, red, and green boxes represent the CK, M, and Bio-M treatment groups, respectively; different p-values denote the significance of differences between treatment groups (p < 0.05 is considered significant).
